# Assessment of periodontitis vaccine using three different bacterial outer membrane vesicles in canine model

**DOI:** 10.1128/msphere.01033-24

**Published:** 2025-03-18

**Authors:** Ryoma Nakao, Takehiro Yamaguchi, Haruka Shibasaki, Jun Saeki, Aoi Takahashi, Ryunosuke Tominaga, Kimihiro Abe, Yukihiro Akeda, Tomoyo Nakagawa-Nakamura, Tomohiko Nishino, Kazuyuki Ishihara, Atsushi Jinno-Oue, Satoshi Inoue

**Affiliations:** 1 Department of Bacteriology I, National Institute of Infectious Diseases, Toyama, Tokyo, Japan; 2 Department of Animal Sciences, Teikyo University of Science, Adachi, Tokyo, Japan; 3 School of Bioscience and Biotechnology, Tokyo University of Technology, Hachioji, Tokyo, Japan; 4 Research Center for Drug and Vaccine Development, National Institute of Infectious Diseases, Toyama, Tokyo, Japan; 5 Department of Microbiology, Tokyo Dental College, Chiyoda, Tokyo, Japan; 6 Bioresource Center, Gunma University Graduate School of Medicine, Maebashi, Gunma, Japan; 7 Department of Veterinary Science, National Institute of Infectious Diseases, Toyama, Tokyo, Japan; University of Wyoming College of Agriculture Life Sciences and Natural Resources, Laramie, Wyoming, USA

**Keywords:** outer membrane vesicles (OMVs), vaccines, adjuvant, periodontal disease, beagle model, *Porphyromonas gingivalis*, *Treponema denticola*: probiotic *Escherichia coli*

## Abstract

Canines frequently develop periodontitis, which is similar and relevant to immunopathology and microbiology of human periodontitis. The aim of this study was to investigate whether bacterial outer membrane vesicle (OMV)-based periodontal vaccines induced humoral immune response in canines from a human vaccine development perspective. *Porphyromonas gingivalis* (Pg) and *Treponema denticola* (Td), two major periodontal pathobionts, were chosen as vaccine targets. Intranasal (IN) immunization with Pg OMVs and Td OMVs strongly elicited humoral immune responses against the two respective species in preparative mouse experiments, particularly when adjuvanted with a probiotic *Escherichia coli* derivative (EcNΔ*flhD*)-derived OMVs. However, in beagles, intranasal immunization with the same Pg/Td/EcNΔ*flhD* OMV vaccine insufficiently elicits humoral immune responses. Nevertheless, the subcutaneous booster with the same OMVs dramatically improved antibody responses in both systemic blood circulation and mucosal sites such as eyes, oral cavity, and upper and lower respiratory tracts. Metagenomic analysis of salivary microbiota revealed that the OMV vaccine might change the microbial composition, while not reducing the number of any periodontal pathobionts at least during the timeframe of the present beagle study. In *in vitro* Pg growth inhibition assay, serum samples from OMV-immunized beagles significantly inhibited growth of the gingipain-deficient strain but not the gingipain-expressing wild-type strain. Taken together, our data offer the trivalent OMV vaccine strategy by IN-prime/SC-boost regimen, which could elicit robust mucosal immune responses, while suggesting the requirement of revised periodontal vaccine regimen toward achievement of sterilizing immunity in the oral cavity.

**IMPORTANCE:**

Bacterial outer-membrane vesicles (OMVs) are attractive for use as novel nanoparticle adjuvants, as well as delivery platforms. Periodontal diseases are the most prevalent oral diseases in humans and have serious health and economic burdens, greatly reducing quality of life. The aim of this study is to investigate the humoral immune responses to an OMV-based periodontal disease vaccine in beagles. The vaccine elicited strong mucosal immune responses when administered to beagles by a four-dose heterologous immunization (IN-IN-IN prime and subcutaneous [SC] boost). The OMV vaccine significantly altered the composition of the microbial community in the oral cavity. These findings suggest the utility of the intranasal (IN) prime followed by the SC boost regimen as a rational option to elicit robust humoral immune responses in canines, and most probably in humans as well. We here discuss the outcomes of beagle experiments, the mechanism behind immunological escape of Pg from host immunity, and a rational perspective toward sterilizing immunity in the oral cavity.

## INTRODUCTION

Periodontitis is characterized as a chronic multifactorial inflammatory disease associated with dysbiotic polymicrobial biofilms in periodontal pockets, and ultimately leads to tooth loss and negatively affects masticatory function, aesthetics, and quality of life. In a study of the global burden of oral health, the age-standardized prevalence of severe periodontitis was 9.8% in 2017, while the number of prevalent cases was 0.8 billion (1). Regarding the etiology of periodontitis, recent metagenomic studies have shown that a wide range of bacterial species are associated with periodontal diseases: not only the well-known “red and orange complexes” species (2) but also newly recognized bacteria such as *Filifactor alocis* (3, 4). Among the red complex bacteria, both *Porphyromonas gingivalis* (Pg) and *Treponema denticola* (Td) are anaerobic Gram-negative periodontal pathobionts, which are associated with oral dysbiosis (5–7), as well as a wide range of extra-oral diseases such as diabetes mellitus, cardiovascular diseases, and cancers (8–14). Several studies have shown the ecological and metabolic relationship and the physical interaction between Pg and Td (15–18), suggesting the synergistic pathogenesis in the oral cavity. The increased evidence implies the clinical relevance and importance of oral microbiota in general health promotion and disease prevention and requires novel regimens to ameliorate oral dysbiosis, such as vaccine development targeting specific periodontal pathobionts.

Regarding experimental animal species for periodontal disease models, mice have been frequently used because of several advantages over other species, e.g., easy handling, low cost, abundance of relevant reagents, and collection of genetically modified animals, and so on. However, it is impossible to examine the vaccine efficacy in rodents since they do not spontaneously develop periodontal disease. In addition, canines live in the same environment as humans develop diseases similar to those of humans (19). It has been reported that several human periodontopathic species including Pg and Td were detected in canines, and likely transmitted from humans to their companion dogs, and vice versa (20). Therefore, canines could be an excellent model to study periodontal disease of humans. In addition, given the high prevalence of periodontal disease in canines (21) and the potential link between periodontal disease and systemic disorders such as cardiovascular, renal, and hepatic diseases (22, 23), a safe and effective preventive vaccine against periodontitis is desired among not only humans but also companion animals. The “One-human-animal-environment Health” perspective can synergistically contribute to the oral health promotions of both humans and companion animals, e.g., a veterinary clinical trial of periodontitis vaccine with companion animals, in anticipation of a future clinical study with human subjects.

Vaccines are the most effective way to combat infectious diseases. We have shown the efficacy and safety of bacterial outer membrane vesicles (OMVs)-based vaccine regimen as a novel vaccine platform candidate (24–27). OMVs are nanometer-scaled proteoliposomes, which carry not only pathogen-specific antigens, but also pathogen-associated molecular patterns such as lipopolysaccharide (LPS), lipoprotein, peptidoglycan, and DNA/RNA to the host immune system, which subsequently evoke immune responses via OMV-host interaction (28, 29). For example, a flagella-deficient probiotic *Escherichia coli* derivative (EcNΔ*flhD*)-derived OMVs have recently been well-characterized as novel nanoparticle adjuvants, as well as delivery platforms (27, 30, 31). In addition, we have shown that intranasal (IN) immunization with Pg OMVs strongly elicited mucosal immune responses in mice and achieved sterilizing immunity in the oral cavity when co-administered with an appropriate mucosal adjuvant, such as a TLR3 agonist, Poly (I:C) (24, 25). Pg OMVs have strong antigenicity; however, OMVs alone exhibit poor self-adjuvanticity in mice (26). In other words, the Pg OMV vaccine required a strong adjuvant to elicit robust Pg-specific immune responses in mice. This may be the same as in canines and primates.

The aim of this study is to investigate the humoral immune responses to OMVs vaccine in beagles. The periodontal disease vaccine composed of trivalent Pg/Td/EcNΔ*flhD*-derived OMVs elicited strong mucosal immune responses when administered to beagles not by a three-dose homologous IN immunization (IN/IN/IN), but by a four-dose heterologous immunization (IN-IN-IN prime and subcutaneous [SC] boost). While our microbial analysis did not detect any vaccine effect against periodontal pathobionts at least during the timeframe of the beagle study, the OMV vaccine altered the composition of the microbial community in the oral cavity. On the other hand, Pg growth assay in the presence of sera of beagles showed that the OMV-immunized serum samples significantly inhibited growth of the gingipain-deficient strain, but not the gingipain-expressing parental strain. We here discuss the outcomes of beagle experiments, the mechanism behind immunological escape of Pg from host immunity, and a rational perspective toward sterilizing immunity in the oral cavity.

## MATERIALS AND METHODS

### Bacterial strains and growth conditions

*Porphyromonas gingivalis* (Pg) strain ATCC 33277 and the isogenic, triple gingipain mutant strain KDP981 (32), and *Porphyromonas gulae* (Pgul) strain ATCC 51700, *Porphyromonas salivosa* (Psal) strain ATCC 49407 were anaerobically grown in brain heart infusion broth (BHI; Becton Dickinson, Sparks, MD, USA) supplemented with hemin and menadione (BHI-HM) or on BHI-HM blood agar plates. *Aggregatibacter actinomycetemcomitans* (Aa) strain Y4 was anaerobically grown in BHI broth or on BHI blood agar plate. *Treponema denticola* (Td) strain ATCC 35405 was anaerobically grown in TYGVS broth containing tryptone (Becton Dickinson), yeast extract (Becton Dickinson), gelatin (Becton Dickinson), volatile fatty acids, and rabbit serum (33). A flagella-deficient derivative of *Escherichia coli* probiotic strain (EcNΔ*flhD*) (30) was grown either in LB broth or on LB agar at 37°C in aerobic conditions.

### Isolation and characterization of OMVs

MVs of all strains of periodontal pathobionts (Pg, Pgul, Aa, and Td) were isolated by PVDF membrane filtration and ultracentrifugation of the bacterial culture supernatants, as described previously (25). MVs of EcNΔ*flhD* were isolated by glycine induction method as described previously (30, 31), in which the MVs were compulsorily induced by extra glycine supplemented in LB broth. Isolated OMVs of all strains were suspended in PBS and analyzed by Bradford assay, field-emission scanning electron microscopy (FE-SEM), and SDS-PAGE, as described previously (30). Particle distribution of OMVs of Pg, Td, and EcNΔ*flhD* OMVs was examined by nano-flow cytometry (nFCM) as described previously (27).

### Endotoxin assay

The *Limulus* assay was performed to compare the endotoxin activities of OMVs among different species, using an Endospecy ES-50M kit (Seikagaku Co., Tokyo, Japan) according to the manufacturers’ instructions, with LPS from *Escherichia coli* O111:B4 (Sigma) as the standard.

### Mouse experiments

**Fig 1:**
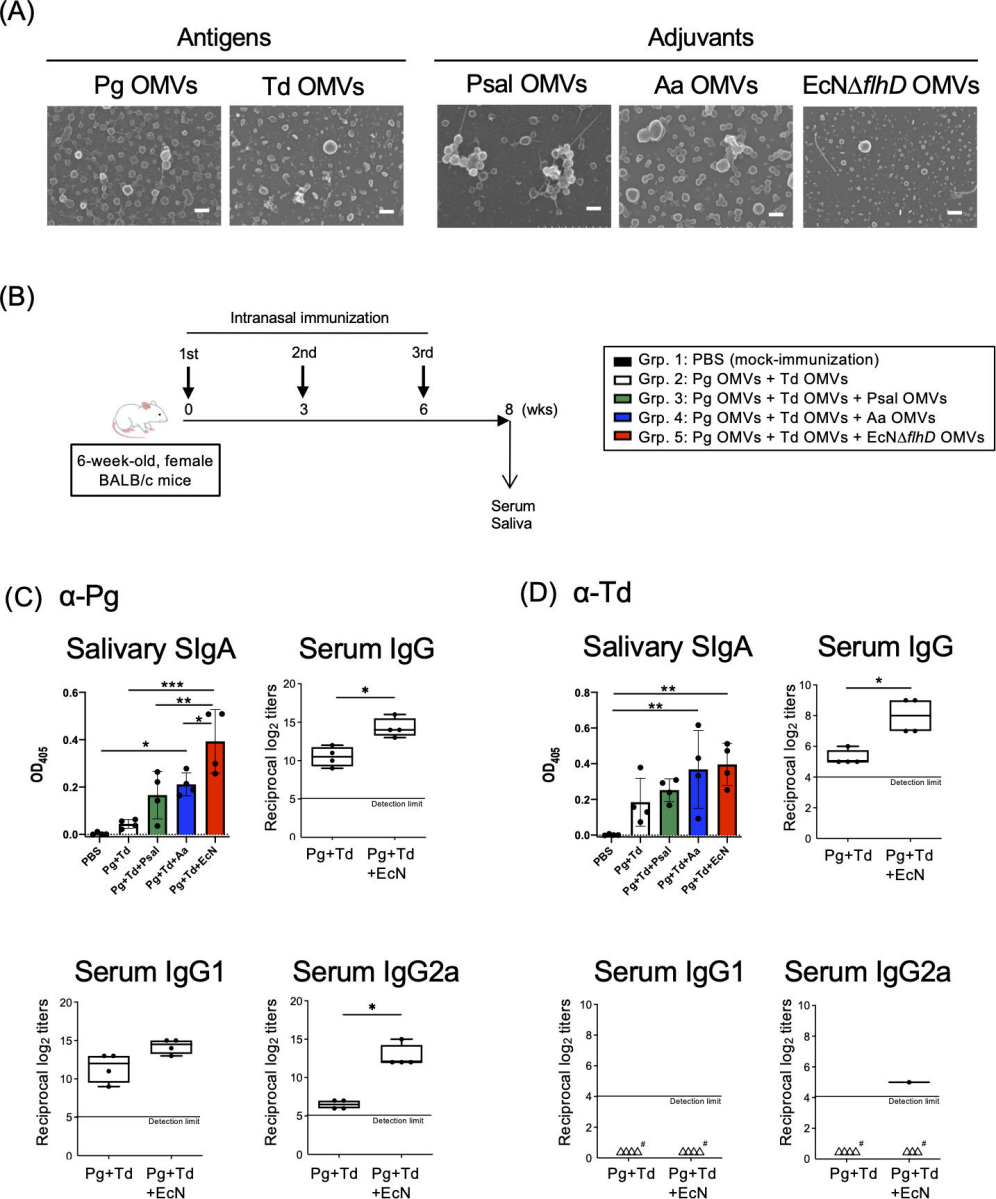
Immunogenicity/adjuvanticity of different bacterial species-derived OMVs in intranasal vaccine model mice. (**A**) Morphology of bacterial OMVs of different origins. In the present study, OMVs of Pg and Td were used as antigen, while those of Psal, Aa, and EcNΔ*flhD* were used as adjuvant. Shown are representative FE-SEM images of OMVs of Pg, Td, Psal, Aa, and EcNΔ*flhD*. Bars represent 100 nm. (**B**) Timeline of immunization. Mucosal adjuvanticity of three different OMVs isolated from Psal, Aa, and EcNΔ*flhD* were compared. Six-week-old female BALB/c mice (*N* = 20) were grouped into five groups with different vaccine formulations shown in the legends and were intranasally immunized three times at weeks 0, 3, and 6. OMV samples of each strain (Pg, Td, Psal, Aa, and EcNΔ*flhD*) were used at the dose of one µg/mouse for intranasal immunization. At 8 weeks, serum and salivary samples were obtained. Pg- and Td-specific antibody responses were analyzed by enzyme-linked immunosorbent assay (ELISA) in which each well was coated with whole cells of Pg and Td, respectively. (**C**) Pg-specific immunoglobulin responses in mice. The level of salivary SIgA production was compared among the five groups (*N* = 4, each) at a single dilution point (1:100). The results are expressed as OD_405_ values (mean ± SD) after a 120 min incubation with AP substrate. One-way analysis of variance (ANOVA) followed by Tukey’s multiple comparison test was used for the statistical analysis. Regarding Pg-specific serum IgG, IgG1, and IgG2a in mice immunized with Pg and Td OMVs (Grp. 2, *N* = 4) and Pg, Td, and EcNΔ*flhD* OMVs (Grp. 5, *N* = 4), the results are expressed as the reciprocal log_2_ of the endpoint dilution. The Mann-Whitney *U*-test was used for the statistical analysis. *P ≤ 0.05, **P ≤ 0.01, ***P ≤ 0.001. (**D**) Td-specific immunoglobulin responses in mice. The level of salivary SIgA production was compared among the five groups (*N* = 4, each) at a single dilution point (1:100). The results are expressed as OD_405_ values (mean ± SD) after a 120 min incubation with AP substrate. One-way ANOVA followed by Tukey’s multiple comparison test was used for the statistical analysis. Regarding Td-specific serum IgG, IgG1, and IgG2a in mice immunized with Pg and Td OMVs (Grp. 2, *N* = 4) and Pg, Td, and EcNΔ*flhD* OMVs (Grp. 5, *N* = 4), the results are expressed as the reciprocal log_2_ of the endpoint dilution. The Mann-Whitney *U*-test was used for the statistical analysis. *P ≤ 0.05, **P ≤ 0.01. ^#^Open triangles indicate the samples under the detection limit (24).

The timeline of IN immunization experiments using BALB/c mice is shown in Fig. 1B.

#### Immunization and ELISA

The procedure of immunization and sample collection using female BALB/c mice aged 6 to 8 weeks (Japan SLC, Inc., Hamamatsu, Japan) is described in recent literature (34). Serum and salivary samples obtained at week 8 were tested for whole-cell enzyme-linked immunosorbent assay (ELISA), using 96-well plates pre-coated with 10 µg/well of the freeze-dried whole cells of a series of periodontal pathobionts (Pg, Td, Pgul, Psal, Aa) and EcNΔ*flhD* (35). Alkaline phosphatase (AP)-labeled goat anti-mouse IgM and IgG (Thermo, San Jose, CA, USA), AP-labeled goat anti-mouse IgA and IgE (Southern Biotech, Birmingham, AL, USA), were used as secondary antibodies at 1:1,000 dilutions. The reactivities were evaluated by chromogenic development using para-nitrophenyl phosphate. Absorbance at 405 nm (OD_405_) was measured to detect enzyme reactions of AP. Regarding Pg- and Td-specific serum IgG, IgG1, and IgG2a, the endpoint titers were also expressed as the reciprocal log_2_ of the last dilution that provided an OD_405_ of 0.05 greater than the background level, which was determined as the average of 2 mice immunized with PBS (mock immunization) in the same ELISA plate.

#### Subcutaneous injection and toxicity assessment

Topical reactogenicity in BALB/c mice following the single SC injection of OMVs was conducted as the toxicity assessment. In brief, the whole back hairs of mice were shaved by an electric hair clipper and hair removal mousse one day before the injection. The OMVs were suspended with PBS containing 0.5% carboxymethyl cellulose sodium salt (CMC, Nakarai, Tokyo, Japan), which suppresses the diffusion of OMVs at the injection site. The amount of OMVs of Pg or Td was subcutaneously injected at the dose of 1 or 5 µg. The volume per injection was 100 µL. The transition of the macroscopic findings of the epithelium after injection was recorded by taking photographs for 1 week after administration.

### Beagle experiments

**Fig 2:**
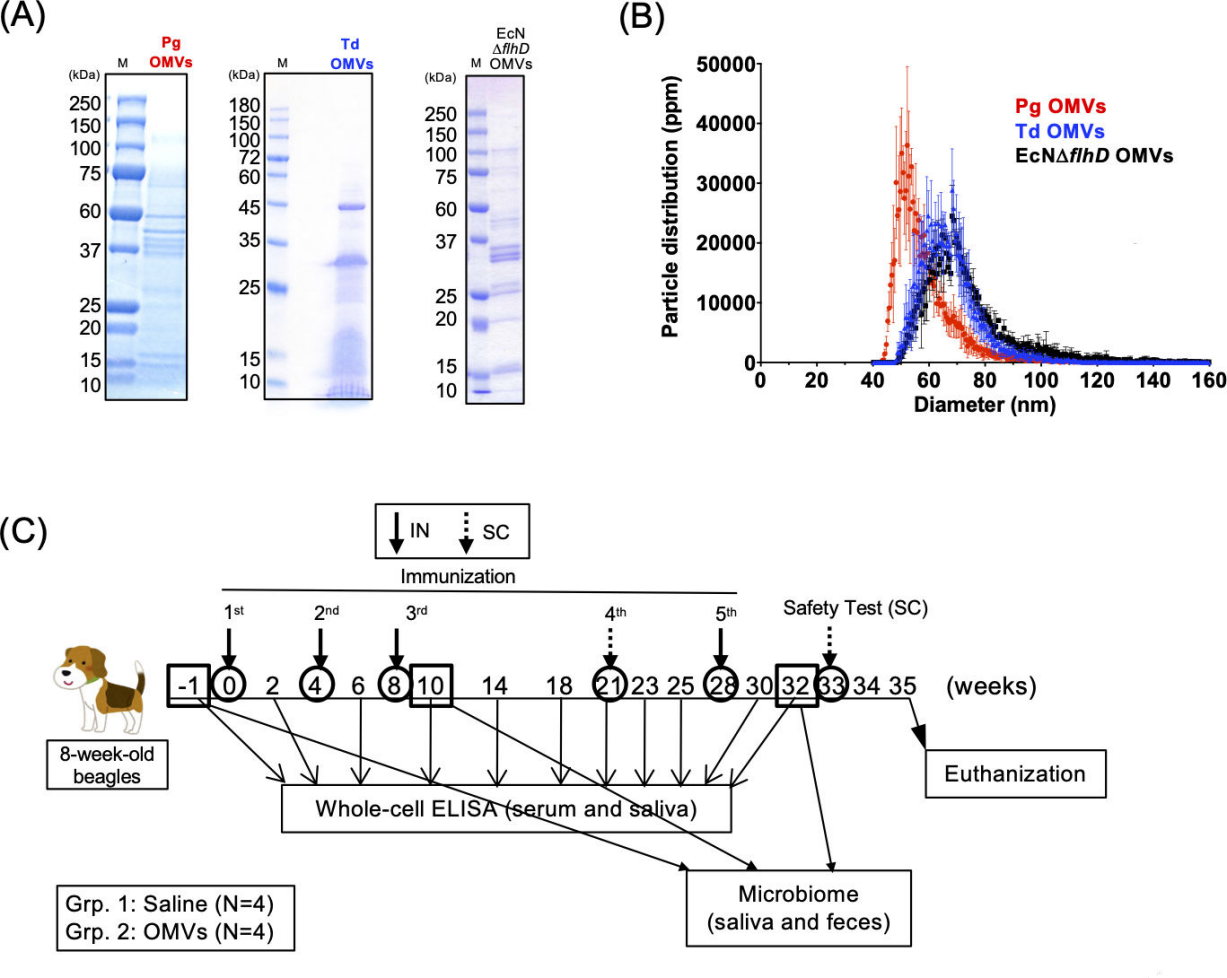
Assessment of Pg/Td/EcNΔ*flhD* OMV vaccine in beagle model. (**A**) Protein profiles of OMVs from Pg, Td, and EcNΔ*flhD*. Pg, Td, and EcNΔ*flhD* OMVs standardized at 4, 10, and 1.5 µg, respectively, were applied to SDS-PAGE. The gels were stained with CBB. ‘M’ represents the molecular weight marker. (**B**) Size distribution of OMVs. Diameter histogram of Pg OMVs (red, *N* = 3), Td OMVs (blue, *N* = 3), and EcNΔ*flhD* OMVs (black, *N* = 3) are shown. Data shown were particle distribution with the mean (fine dots) and SD (vertical bars) of these independent data sets. (**C**) Vaccine regimen in beagles. Time points of immunization/sample collection (weeks after first immunization) are shown on the timeline. Eight-week-old female (*N* = 4) and male (*N* = 4) beagles were divided into saline-administration control group (saline; female *N* = 2, male *N* = 2) and Pg/Td/EcNΔ*flhD* OMV vaccine-administration group (OMVs: female *N* = 2, male *N* = 2). All beagles were intranasally (IN) immunized three times at weeks 0, 4, 8, and 28, and subcutaneously (SC) immunized once at week 21. The IN dose was 50 µg (first, second, and third immunization) or 200 µg (fourth immunization) of each OMV sample obtained from Pg, Td, and EcNΔ*flhD* strains. The SC dose was 250 µg of each OMV sample. Serum and saliva samples for ELISA were collected from week (−1) to week 32 at 2–4 week intervals. Saliva and feces samples for microbiome were collected at week (−1), 10, and 32. After sample collection at week 32, general and topical conditions after SC injection of Pg/Td/EcNΔ*flhD* OMV vaccine were also observed at week 33. In the safety assessment study, two beagles in the OMV-immunized group (female: *N* = 1, male: *N* = 1) were SC administered at low dose (30 µg of each Pg/Td/EcNΔ*flhD* OMV sample/beagle), while another two beagles in the OMV-immunized group (female: *N* = 1, male: *N* = 1) were administered at moderate doses (150 µg of each Pg/Td/EcNΔ*flhD* OMVs/beagle). All beagles were euthanized at week 35.

Eight beagles aged 7 weeks were purchased from Japan SLC, Inc. The timeline of the beagle immunization experiment and safety assessment is shown in Fig. 2C.

#### Immunization and ELISA

The trivalent OMV vaccine or saline control was administered to beagles via the SC or IN route. The SC injection was performed with OMVs in saline or saline control at the back of the neck. The SC dose was 250 µg of each OMV sample. The volume was 500 µL/injection. For the IN administration, mice were immunized by dropping saline or saline containing the OMVs into each nostril. The IN dose was 50 µg (first, second, and third immunization) or 200 µg (fourth immunization) of each OMV sample. The volume was in total 200 µL. The reactivities of serum and saliva samples of beagles to target bacteria were examined by whole-cell ELISA using 96-well plates pre-coated with 10 µg/well of the freeze-dried whole cells of the respective bacterial strain, as described previously (35). Goat anti-canine IgM, IgG, IgA, and IgE (Thermo) were labeled with AP by Alkaline Phosphatase Labeling Kit-NH2 (Dojindo, Kumamoto, Japan). The AP-labeled secondary antibodies against canine immunoglobulins were used at 1:1,000 dilution. The reactivities were evaluated by chromogenic development using para-nitrophenyl phosphate. Absorbance at 405 nm (OD_405_) was measured to detect enzyme reactions of AP. Regarding Pg-, Pgul-, and Td-specific antibody responses (serum IgG, bronchoalveolar lavage fluid [BALF] IgG, nasal swab IgA, and oral swab IgA), the responsiveness was expressed as the dose-response curve (OD_405_) generated by twofold serial dilutions of these samples.

#### Toxicity assessment following subcutaneous injection of OMVs

For safety assessment, the doses of trivalent OMVs vaccine subcutaneously injected to a beagle were from 4.0 to 27.2 µg (per kg of body weight). Evaluation of general conditions were conducted for a week after injection, according to criteria including five symptoms: (i) fever not less than 39°C, (ii) cornea hyperemia, (iii) nausea or vomiting, (iv) hypersalivation, and (v) general malaise.

#### Sample collection and euthanization

Each protocol listed below is described in the order of samples collected.

##### Blood draw

Approximately 2 mL of whole blood was collected from the lateral saphenous vein after shaving hairs.

##### Saliva collection

Saliva was collected by using a 125-mm-length cotton roll (# 5001.06; Salimetrics LLC, Carlsbad, CA) and saved at −20°C. For DNA extraction, an aliquot of saliva (100 µL) was centrifuged at 15,000 rpm for 5 min. The pellet after centrifugation was washed once with PBS and centrifuged at 15,000 × *g* for 5 min. The pellet after centrifugation was used for DNA isolation.

##### Feces pretreatment for DNA extraction

Fifty milligram of fresh feces in 500 µL of PBS was vigorously mixed and dispersed by a vortex mixer. After short centrifugation at 5,000 rpm for 20 s, the supernatant was collected, saved at −20°C, and used for ELISA. The aliquot of the feces supernatant was further centrifuged at 15,000 × *g* for 5 min. The pellet after centrifugation was used for DNA isolation.

##### Cerumen collection

Cerumen was collected with paper-made, small ear buds for infants of humans.

##### Euthanization

Beagles were euthanized with intravenously injected pentobarbital at 50 µg/kg of body weight (BW), under sedation with intramuscularly injected medetomidine at 50 µg/kg of BW.

##### Eye wash collection

Eye wash was collected from mice immediately after euthanization. One hundred microliters of saline were put on the ocular surface and pipetted 10 times without touching the ocular surface or skin around the eyes. Both eyes were washed with saline once, and eventually a total of 200 µL of eye wash (100 µL/eye) was pooled together in a tube and saved at −20°C.

##### Nasopharyngeal swab

A nasopharyngeal swab was collected using a cotton swab. The mucosal surface of the nasal cavity was scrubbed by moving the cotton swab back and forth for 10 times from each nostril. The solution of cotton tip was transferred to an Eppendorf tube prefilled with 500 µL and saved at −20°C.

##### Oral swab

An oral swab was collected using a cotton swab. Oral mucosa at upper right and upper left gingival margins of molar teeth was scrubbed by moving the cotton swab back and forth for 10 times. The solution of cotton tip was transferred to an Eppendorf tube prefilled with 500 µL and saved at −20°C.

##### Rectal swab

A rectal swab was collected using a cotton swab. The mucosal surface of the rectum was scrubbed by moving the cotton swab back and forth for 10 times from the anus. The solution of cotton tip was transferred to an Eppendorf tube prefilled with 500 µL and saved at −20°C for ELISA.

##### Penis swab

A penis swab was collected using a cotton swab. The whole surface of the glans-penis was scrubbed by wetting the swab. The solution of the cotton tip was transferred to an Eppendorf tube prefilled with 500 µL and saved at −20°C.

##### Vaginal swab

A vaginal swab was collected using a cotton swab. Vaginal mucosa was scrubbed by wetting the swab. The solution of the cotton tip was transferred to an Eppendorf tube prefilled with 500 µL and saved at −20°C.

##### Bronchoalveolar lavage fluid

BALF was collected using a laryngoscope and a 14- or 16-Fr Foley catheter. Prior to insertion of the catheter, the distance from the neck to the tracheal carina was measured using a long chopstick. Under the guidance of the laryngoscope, the catheter was inserted and placed until the end of the catheter in the right main bronchi by 8 cm distal from the tracheal carina. Immobilize the catheter by pumping the balloon with 3 mL sterilized water. Then bronchoalveolar was washed three times with 20 mL of saline. The solution was collected as BALF and saved at −20°C.

### Bacterial DNA extraction

Bacterial DNA was extracted from both saliva and feces of beagles at weeks −1, 10, and 32 by a protease/RNase-based purification method using DNeasy Blood and Tissue Kit, according to the manufacturer’s instructions.

### Real-time PCR

To quantify the number of Pg, Pgul, Td, Fn, and Tf in beagles’ saliva, TaqMan-based real-time PCR assays were performed by using a LightCycler 96 (Roche Diagnostics, Basel, Switzerland) with a GeneAce Probe qPCR Mix II (Nippon Gene Co., Ltd., Tokyo, Japan). The primers and probes to detect each gene target are shown in Table S1.

### Metagenomic analysis of the microbiome

Salivary and fecal microbiota were examined by a 16S rRNA amplicon-based metagenomic analysis. In brief, two-step PCR was performed to obtain the V3/V4 region sequence libraries from purified DNA samples. The first PCR was performed using PrimeStar HS (Takara Bio. Shiga, Japan) with 341F (5′-ACACTCTTTCCCTACACGACGCTCTTCCGATCT-NNNNN-CCTACGGGNGGCWGCAG-3′) and 805R (5′-GTGACTGGAGTTCAGACGTGTGCTCTTCCGATCT-NNNNN-GACTACHVGGGTATCTAATCC-3′). After PCR product purification using AMPure XP (Beckman Coulter, Indianapolis, IN), the second PCR was performed using ExTaq HS (Takara Bio) with second forward primer (5′-AATGATACGGCGACCACCGAGATCTACAC-Index2-ACACTCTTTCCCTACACGACGC-3′) and second reverse primer (5′-CAAGCAGAAGACGGCATACGAGAT-Index1-GTGACTGGAGTTCAGACGTGTG-3′) to add barcode sequences for Illumina sequencing. The prepared libraries were subjected to sequencing using a MiSeq Reagent Kit v3 (Illumina, San Diego, CA) on a MiSeq (Illumina) at Bioengineering Lab (Kanagawa, Japan). Sequence data processing, operational taxonomic unit (OTU) definition, and taxonomic assignment were performed using the Greengenes database and QIIME2 (36). The alpha-diversity (Faith’s, observed species, and Shannon’s phylogenetic diversity indices) was determined and analyzed using the Mann-Whitney *U*-test. The beta-diversity was estimated using the weighted UniFrac metric and was visualized via principal coordinate analysis with the difference between saline and OMVs groups tested via permutational analysis of variance (PERMANOVA). The differential abundance of the OTUs at the different taxonomic levels of organisms between OMV-immunized and saline control beagles was analyzed using the linear discriminant analysis effect size (LEfSe) method (37).

### Serum sensitivity assay

Sensitivity of ATCC 33277 and KDP981 to serum samples of beagles was conducted by a Pg growth inhibition assay using 96-well plates (38), with some modifications. Briefly, a 2-day culture of ATCC 33277 and KDP981 was standardized at OD_600_ = 0.025 with BHI-HM broth. Beagle serum samples were heated at 56°C for 30 min to inactivate the serum complement, then were added to the bacterial suspension at the concentration of 2.5%. Baby rabbit complement (#CL3441SR, Cedarlane, Burlington, Ontario, Canada) was unheated or heated at 56°C for 30 min to inactivate the complement, then was added to the bacterial suspension at the concentration of 2.5%. The final reaction volume of 100 µL. After the samples were incubated for 48 h at 37°C in the anaerobic chamber, the absorbance was measured as the OD_600_ value using a plate reader.

### Statistical analysis

Statistical analysis was performed using the Mann-Whitney *U*-test or one-way analysis of variance (ANOVA) followed by the Dunnett’s multiple comparison test in all experiments, except LEfSE analysis. In LEfSE, a statistical test was performed in combination with the Mann-Whitney *U*-test and ANOVA followed by the Kruskal-Wallis test.

## RESULTS

### Intranasal trivalent Pg/Td/EcNΔ*flhD* OMV vaccine in mice

In the present study, OMVs of Pg and Td were used as antigens, while OMVs of Psal, Aa, or EcNΔ*flhD* (30) were used as adjuvant candidates (Fig. 1A). In FE-SEM analysis, all these purified OMVs were observed as nanometer-scaled spherical structures ranging from 40 to 100 nm in diameter (Fig. 1A; Fig. S1). The surface appearance of OMVs varied among individuals (Fig. 1A; Fig. S1). The OMV surfaces of Pg, Pgul, and Psal were found to be rougher than those of others (Td, Aa, EcNΔ*flhD*) by our improved FE-SEM analysis with sub-nanometer resolution (27), (Fig. S1).

As shown in the timeline of mouse model experiment (Fig. 1B), humoral immune responses after vaccinations with different OMV combinations were evaluated by whole-cell ELISAs of serum and salivary samples (Fig. 1C and D; Fig. S2). Whereas, the IN immunization with Pg/Td OMVs alone failed to induce sufficient antibody production against the respective pathogens, the IN immunization with Pg/Td OMVs adjuvanted with Aa OMVs, Psal OMVs, or EcNΔ*flhD* OMVs strongly elicited Pg- and Td-specific antibodies. In particular, the most potent humoral immune responses to Pg were induced in mice adjuvanted with the EcNΔ*flhD* OMVs. Of note, Pgul whole-cell ELISA data showed that the trivalent Pg/Td/EcNΔ*flhD* OMV vaccine strongly induced cross-reactive antibodies to Pgul. The results of Pg whole-cell ELISA (Fig. 1C and D; Fig. S2) were very similar to those of Pgul whole-cell ELISA (Fig. S2). On the other hand, while Psal-specific antibody was detected only in the Pg/Td/Psal OMV vaccine group (Fig. S2), suggesting that Pg is antigenically similar to Pgul, but is distinct from Psal.

### IN trivalent Pg/Td/EcNΔ*flhD* OMV vaccine in beagles

**Fig 3:**
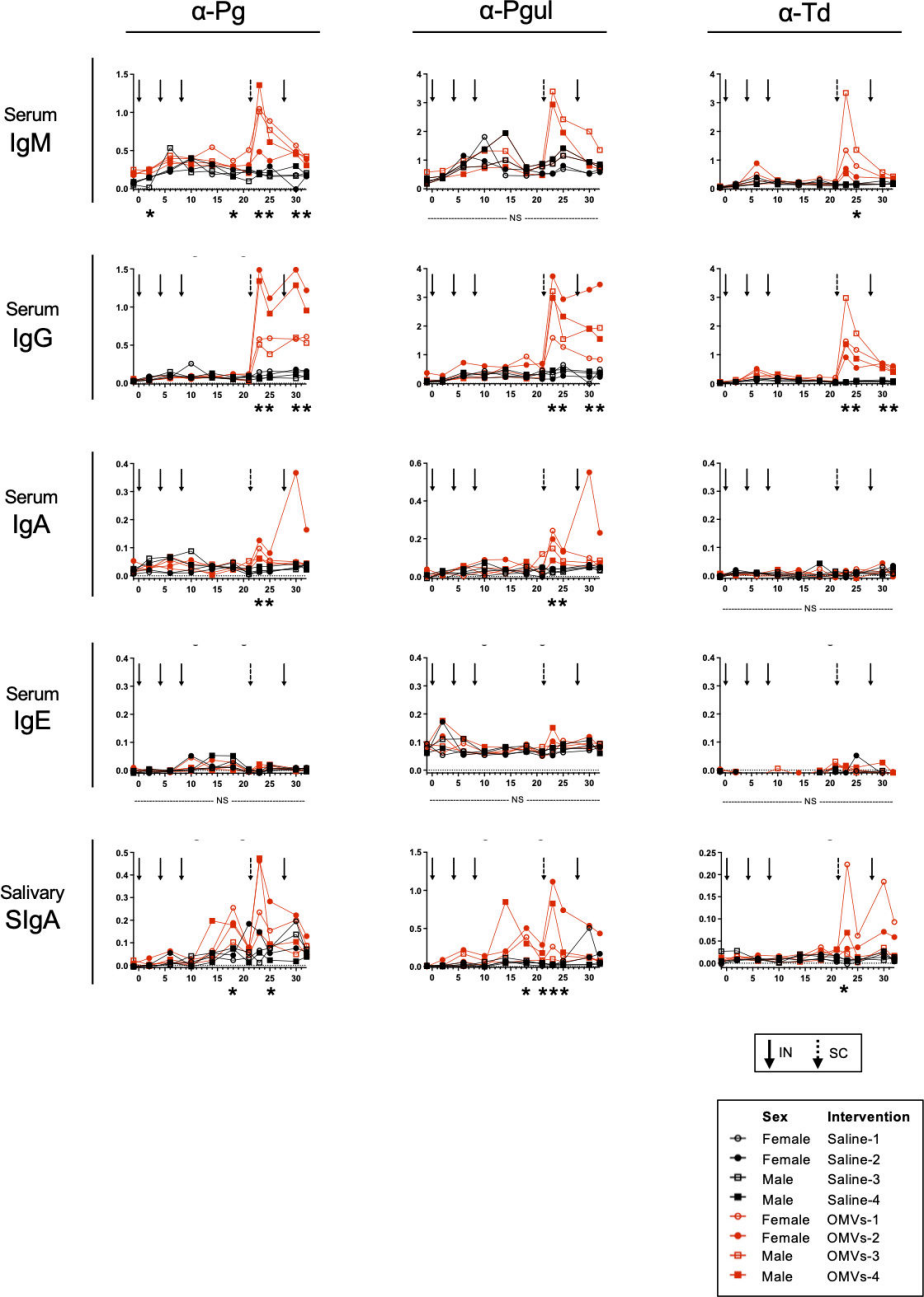
Transition of Pg, Pgul, and Td-specific antibody responses in beagles. Pg-, Pgul, and Td-specific antibody responses (serum IgM, IgG, IgA, IgE, and salivary SIgA) were examined at different time points by ELISA coated with whole cells of Pg, Pgul, and Td. To examine serum IgM and IgG, samples were used at 1:1,000 dilution. To examine serum IgA and IgE, and salivary SIgA, samples were used at 1:100 dilution. The results are expressed as OD_405_ values (mean ± SD) after incubation with AP substrate. Asterisks shown beneath the *X*-axis denote statistically significant difference (*P* < 0.05) when the values were compared between saline (*N* = 4) and OMV groups (*N* = 4) at the same time point. NS, no statistically significant difference. The Mann-Whitney *U*-test was used for the statistical analysis. Arrows and dotted-line arrows denote the time points of intranasal and subcutaneous injections.

**Fig 4:**
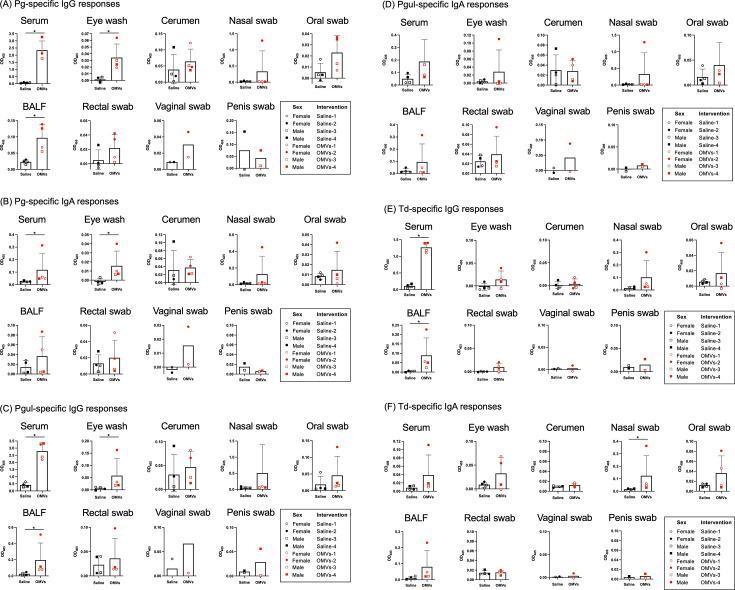
Pg, Pgul, and Td-specific antibody responses in beagles after euthanization Pg-, Pgul-, and Td-specific antibody responses were examined by ELISA coated with whole cells of the respective bacteria. IgG responses to Pg, Pgul, and Td are shown in panels **A**, **C**, and **E**, respectively. IgA responses to Pg, Pgul, and Td are shown in panels **B**, **D**, and **F**, respectively. Tested were samples obtained from different organs at euthanization (serum, eye wash, cerumen, nasal swab, oral swab, BALF, rectal swab, vaginal swab, and penis swab). To examine IgG responses, all those samples were used at 1:1,000 dilution. To examine IgA responses, all those samples were used at 1:100 dilution. The results are expressed as OD_405_ values after incubation with AP substrate. Asterisks denoted statistically significant difference (*P* < 0.05) when compared between saline (*N* = 4) and OMV groups (*N* = 4). Mann-Whitney *U*-test was used for the statistical analysis. Note that the data of vaginal and penis swabs are shown as the results of two beagles (*N* = 2) in each group, respectively.

Based on the findings of humoral immune responses in mice, the Pg/Td/EcNΔ*flhD* OMV vaccine was chosen and used for beagle experiments. Results of nFCM analysis showed that the diameter/particle distribution varied but was less than 100 nm (Fig. 2B), which was in good agreement with observation by FE-SEM in this study (Fig. 1A; Fig. S1) and our recent studies (26, 27). The protein profile of Td OMVs showed two distinct major bands at around 45 and 30 kDa (Fig. 2A). The smear signals were also observed at lower molecular weight at less than 20 kDa (Fig. 2A), probably due to the self-protein degradation by dentilisin, a chymotrypsin-like protease of Td (39). The trivalent Pg/Td/EcNΔ*flhD* OMVs were used as a vaccine candidate for periodontal disease in beagle model experiments (Fig. 2C). Production of Pg- and Td-specific antibodies was examined at different time points during the vaccine regimen (Fig. 3). In the first half of the timeframe (~week 20), homologous 3-dose IN immunization was found not to elicit humoral immune responses very well (Fig. 3), which was in stark contrast with the responses in mice (Fig. 2). On the other hand, subcutaneous boost (fourth immunization) at week 21 with the same OMV vaccine dramatically improved antibody production against Pg and Td in both systemic blood circulation and oral cavity (Fig. 3). Pg-, Pgul-, and Td-specific antibodies were detected at some other mucosa of the OMV-immunized beagles when euthanized at week 34 (Fig. 4; Fig. S3). The levels of Pg- and Pgul-specific IgG in serum, eye wash, and BALF (Fig. 4A and C), Pg-specific IgA in serum and eye wash (Fig. 4B), Td-specific IgG in serum and BALF (Fig. 4E), and Td-specific IgA in nasal swab (Fig. 4F) were significantly higher in OMVs-immunized beagles than saline control beagles.

### Alteration of salivary microbiome by vaccination of OMVs

**Fig 5:**
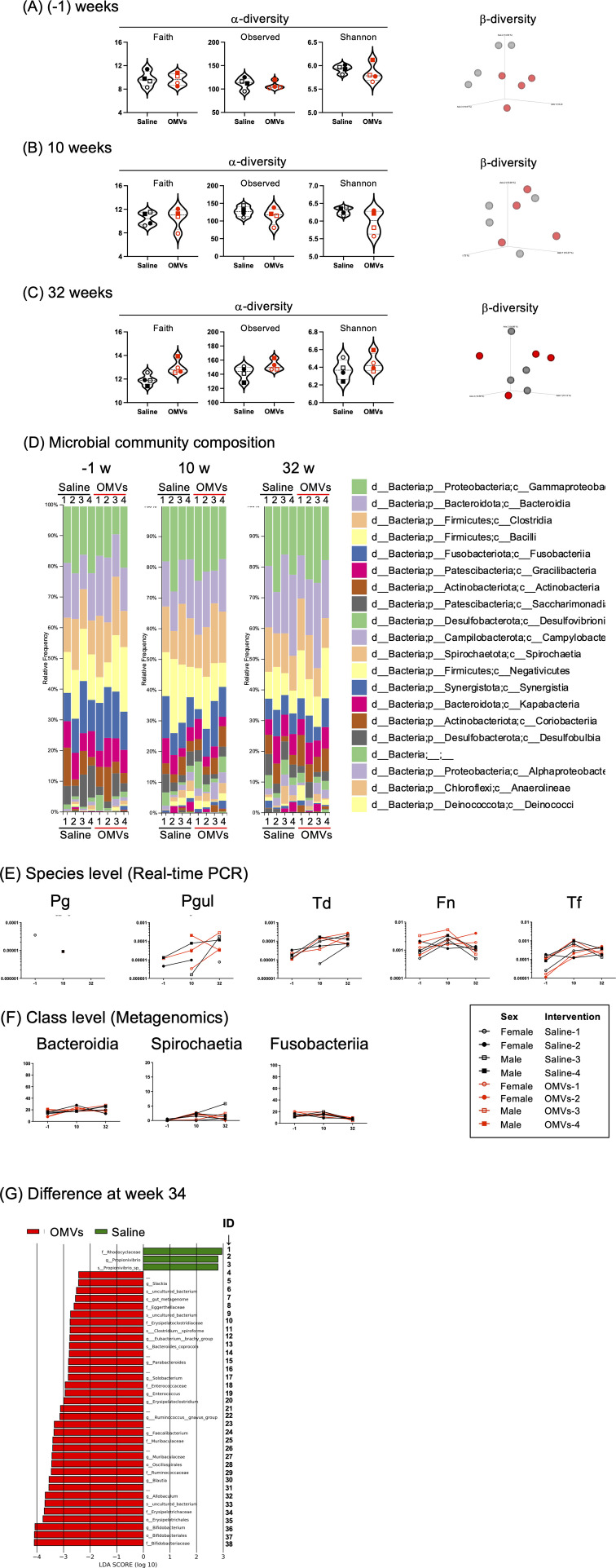
Microbial community in saliva. (A, B, and C) Comparison of the alpha-diversity and beta-diversity between saline and OMVs groups at weeks (−1) (**A**), 10 (**B**), and 32 (**C**). The alpha-diversity indices (Faith phylogenetic diversity [Faith], observed features [Observed], and Shannon) are shown as violin plots and compared using Mann-Whitney *U*-test. The beta-diversity index is visualized via principal coordinate analysis with differences between the two groups and tested with PERMANOVA. (**D**) Microbial distribution. Shown are compositions of the microbial community in saliva at three different time points: weeks (−1), 10, and 32. Data are shown as % of each microbial composition at the class level. (**E**) Transition of the numbers of Pg, Pgul, Td, Fn, and Tf at three different time points: weeks (−1), 10, and 32. Each bacterial number was quantified by species-specific real-time PCR. After normalization by the copy number of universal 16S rRNA gene, the data of each species-specific gene are plotted for each beagle subject and connected by a line. Sex and intervention with ID number are indicated in the table at lower right. (**F**) Transition of the ratio (%) of three major classes (Bacteroidia, Spirochaetia, and Fusobacteriia) at three different time points: weeks (−1), 10, and 32. Data are plotted for each beagle subject and connected by a line. Sex and intervention with ID number are indicated in the table. (**G**) Changes in the relative abundances of the taxonomic compositions of the microbial community at the species and higher levels. The population shown as green or red bar significantly increased in the saline or OMVs group, respectively. Abbreviations: o, order; f, family; g, genus. No name after the character “_” denotes a taxonomic unit that has been identified, but is still unnamed. The ID of OTUs was denoted on the left side. See also the detail in Table S2; Fig. S4.

To examine whether the OMV vaccine modulates the microbial community in saliva of beagles, total bacterial DNA in saliva was analyzed by a metagenomic approach based on 16S rRNA amplicon analysis. The V3-V4 hypervariable region of the 16 S rRNA gene was sequenced to identify the diversity (Fig. 5A through C) and salivary microbial composition (Fig. 5D) in OMVs and saline control beagles at weeks −1, 10, and 32, respectively. In the alpha diversity analysis, which evaluated the diversity within microbiota, the Faith, observed species, and Shannon indices were calculated to assess the microbiota in saliva between saline and OMV groups at the different time points (Fig. 5A through C). The beta diversity was assessed by using the weighted UniFrac metric to examine the similarity or dissimilarity of the microbial community between saline and OMV groups at the different time points (Fig. 5A through C). The data showed no significant difference in both alpha and beta diversities between the groups at each time point. Taxonomic composition analysis at the class level was shown in Fig. 5D. The results of both real-time PCR and microbiota survey showed that there is no significant reduction in the number of all tested periodontal pathobionts between the saline beagles and OMVs-vaccinated beagles at every time point, week −1, 10, and 32 (Fig. 5E). The composition analysis at class level showed that there was no significant difference in the ratio of Bacteroidia, Spirochaetia, and Fusobacteriia between the saline and OMV groups (Fig. 5F. However, in total, 3 and 35 taxonomic components were significantly increased in their relative abundances in the saline and OMV groups, respectively, by LEfSe analysis (37) (Fig. 5G; Fig. S4; Table S2). The components increased in saliva of the saline or OMVs groups were shown in Fig. S4A and B, respectively. In contrast, analysis of fecal microbiota showed that only two taxonomic components were significantly changed in their relative abundances in the saline control group (Fig. S5). Taken together, the efficiency of the OMV vaccine against periodontal pathobionts was not detected at least during the short timeframe of the present beagle study; however, the OMV vaccine seemed to alter microbial composition in the oral cavity rather than in the gastrointestinal tract.

### Safety of doses of OMVs used for vaccination in mice and beagles

For the estimation of endotoxin in the OMV vaccine, the *Limulus* assay was performed using three biological replicates of EcNΔ*flhD* OMVs, Pg OMVs, and Td OMVs. In our recent report (26), the endotoxin activity of Aa OMVs was 70.0 ± 55.1 EU/ng. The central nervous system safety of Aa OMVs (1 µg) and Pg OMVs (1 µg) has already been confirmed in the intracerebral injection mouse model (24, 26). In the present study, the endotoxin activity of EcNΔ*flhD* OMVs was found to be 36.5 ± 31.4 (mean ± SD) EU/ng (protein equivalent), which was approximately twofold lower than that of Aa Y4 OMVs (26). The activity of Pg OMVs (0.226 ± 0.163 EU/ng) was further lower than that of EcNΔ*flhD* OMVs. Surprisingly, Td OMVs (0.00139 ± 0.000407 EU/ng) showed more than 100- or 10,000-fold lower activity as compared to that of Pg OMVs or EcNΔ*flhD* OMVs, respectively. Furthermore, for the vaccine safety assessment, the reactogenicity of OMVs with different doses was evaluated in BALB/c mice and beagles. In the BALB/c mice model, the topical reaction after subcutaneous injection with OMVs was examined. The results showed that no reactogenicity was observed in all OMVs used in this study when the injected dose was at least not more than 1 µg (data not shown), which is equivalent to the dose used for intranasal vaccine. In beagles, changes in the general conditions were scored (Table 1). The data showed that no adverse reaction occurred when the dose was used less than 4.7 µg/kg of body weight (BW), although more than 12.1 µg/kg of BW caused several symptoms such as fever (>39°C), nausea or vomiting, hypersalivation, and general malaise. All symptoms disappeared and recovered in all beagles by day 4 (Fig. S6).

**TABLE 1:** Adverse reaction record after subcutaneous administration with OMVs at different doses

Interventions	Subject information: sex, age, BW, dose^*a*^	Symptoms	Day 0	Day 1	Day 2	Day 3
Low dose-1 (each 50 µg of OMVs, protein equivalent) (OMVs-1)	Female, 42 weeks old. 10.6 kg, 4.7 µg/kg of BW	Fever (>39ºC)	(−)	(−)	(−)	(−)
		Cornea hyperemia	(−)	(−)	(−)	(−)
		Nausea or vomiting	(−)	(−)	(−)	(−)
		Hypersalivation	(−)	(−)	(−)	(−)
		General malaise	(−)	(−)	(−)	(−)
Low dose-2 (each 50 µg of OMVs, protein equivalent) (OMVs-3)	Male, 42 weeks old, 12.4 kg, 4.0 µg/kg of BW	Fever (>39ºC)	(−)	(−)	(−)	(−)
		Cornea hyperemia	(−)	(−)	(−)	(−)
		Nausea or vomiting	(−)	(−)	(−)	(−)
		Hypersalivation	(−)	(−)	(−)	(−)
		General malaise	(−)	(−)	(−)	(−)
Moderate dose-1 (each 150 µg of OMVs, protein equivalent) (OMVs-2)	Female, 42 weeks old, 11.2 kg, 13.4 µg/kg of BW	Fever (>39ºC)	(+)	(−)	(−)	(−)
		Cornea hyperemia	(−)	(−)	(−)	(−)
		Nausea or vomiting	(+)	(−)	(−)	(−)
		Hypersalivation	(+)	(−)	(−)	(−)
		General malaise	(+)	(−)	(−)	(−)
Moderate dose-2 (each 150 µg of OMVs, protein equivalent) (OMVs-4)	Male, 42 weeks old, 12.4 kg, 12.1 µg/kg of BW	Fever (>39ºC)	(+)	(−)	(−)	(−)
		Cornea hyperemia	(+)	(−)	(−)	(−)
		Nausea or vomiting	(+)	(−)	(−)	(−)
		Hypersalivation	(−)	(−)	(−)	(−)
		General malaise	(+)	(−)	(−)	(−)
High dose-1 (each 250 µg of OMVs, protein equivalent) (OMVs-1)	Female, 29 weeks old, 9.5 kg, 26.3 µg/kg of BW	Fever (>39ºC)	(+)	(+)	(+)	(−)
		Cornea hyperemia	(+)	(+)	(−)	(−)
		Nausea or vomiting	(+)	(+)	(+)	(−)
		Hypersalivation	(+)	(+)	(−)	(−)
		General malaise	(+)	(+)	(+)	(±)
High dose-2 (each 250 µg of OMVs, protein equivalent) (OMVs-2)	Female, 29 weeks old, 9.5 kg, 26.3 µg/kg of BW	Fever (>39 ºC)	(+)	(+)	(+)	(−)
		Cornea hyperemia	(+)	(+)	(−)	(−)
		Nausea or vomiting	(+)	(+)	(+)	(−)
		Hypersalivation	(+)	(+)	(−)	(−)
		General malaise	(+)	(+)	(+)	(±)
High dose-3 (each 250 µg of OMVs, protein equivalent) (OMVs-3)	Male, 29 weeks old), 9.2 kg, 27.2 µg/kg of BW	Fever (>39ºC)	(+)	(+)	(−)	(−)
		Cornea hyperemia	(+)	(+)	(−)	(−)
		Nausea or vomiting	(+)	(+)	(−)	(−)
		Hypersalivation	(+)	(+)	(−)	(−)
		General malaise	(+)	(+)	(−)	(−)
High dose-3 (each 250 µg of OMVs, protein equivalent) (OMVs-4)	Male, 29 weeks old, 9.2 kg, 27.2 µg/kg of BW	Fever (>39ºC)	(+)	(+)	(+)	(−)
		Cornea hyperemia	(−)	(−)	(−)	(−)
		Nausea or vomiting	(−)	(−)	(−)	(−)
		Hypersalivation	(−)	(−)	(−)	(−)
		General malaise	(−)	(−)	(−)	(−)

^*a*^ BW, body weight.

### Pg immunologically escapes from Pg-specific antibodies of beagles vaccinated with OMVs in a gingipain-dependent manner

**Fig 6:**
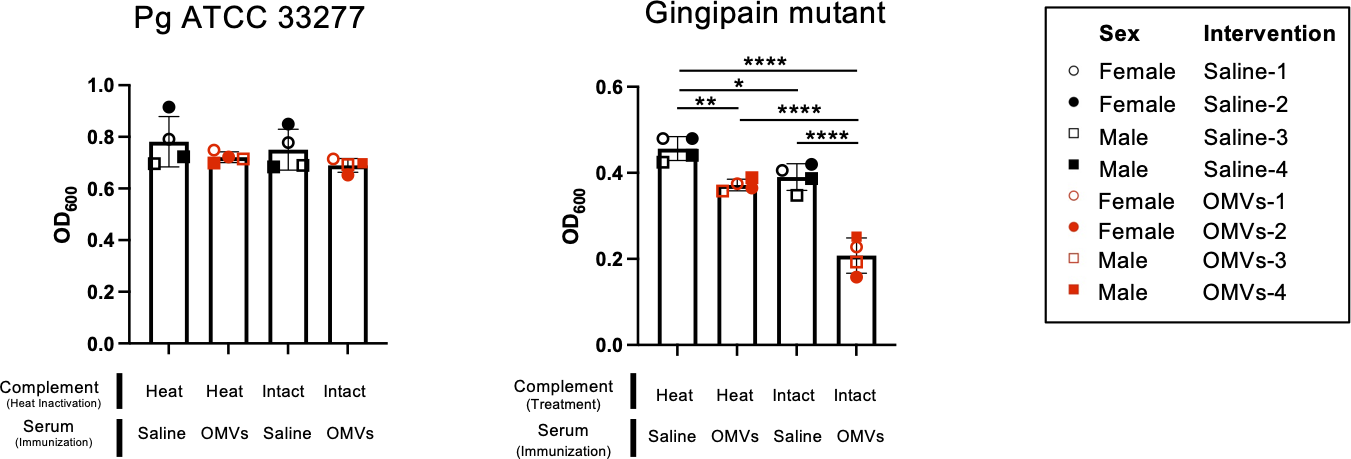
Gingipains protect Pg growth from *in vitro* killing by beagle sera Pg wild-type strain (ATCC 33277, left panel) and the gingipains triple mutant strains (right panel) were grown in the presence of serum samples (2.5%/vol) of beagle mock-immunized with saline (*N* = 4, black dots) or immunized with OMV vaccines (*N* = 4, red dots). All serum samples were inactivated at 56°C for 30 min. The influence of rabbit complement (2.5%/vol) was also assessed by adding heat inactivated complement (heat) and non-treated complement (intact) to the bacterial culture. *Y*-axes show the absorbance of bacterial culture (OD_600_) at 48 h. Data are expressed as means ± SD; *n* = 4. One-way ANOVA followed by the Dunnett’s multiple comparison test was used for the statistical analysis. *P < 0.05, **P < 0.01, ****P < 0.0001.

Finally, we performed Pg growth inhibition assays in the presence of beagle sera to examine whether Pg-specific antibodies of OMV-immunized beagles inhibit Pg growth *in vitro*. Neither the saline beagles’ sera nor the OMV-immunized beagles’ sera inhibited the growth of the wild-type strain (Pg ATCC 33277, Fig. 6). The same experiment was performed with the isogenic gingipain-deficient strain (Gingipain mutant, Fig. 6), in place of the wild-type strain. Notably, serum samples from OMV-immunized beagles significantly inhibited growth of the gingipain-deficient strain as compared to the saline beagles’ sera or the heat-inactivated OMV-immunized sera (Gingipain mutant, Fig. 6). Especially, the inhibition activities of OMV-immunized sera were significantly enhanced in the presence of intact baby rabbit complement (Gingipain mutant, Fig. 6).

## DISCUSSION

Several reports have shown that nanoparticles are expected to induce an immune response as vaccine carriers (40), since nanoparticle vaccines in the range of from 10 to 100 nm in diameter drain from interstitial tissue into lymphatic vessels, where they can accumulate within local lymph nodes (41). In the morphological evaluation, both FE-SEM and nFCM analyses showed that the diameters of OMVs used in this study varied among individual origins (Fig. 1A and 2B). The main peaks of OMVs of Pg, Td, and EcNΔ*flhD* were approximately 50, 65, and 75 nm in diameter, respectively (Fig. 2B). So, the sizes were in the range of less than 100 nm. As we expected, the strong immunogenicity of the trivalent Pg/Td/EcNΔ*flhD* OMV vaccine was confirmed in both the BALB/c mouse and beagle models (Fig. 1 to 3).

Pg OMVs have already been well-characterized as not only a potent mucosal immunogen (25) but also a stable antigen-delivery system (24), while a weak mucosal adjuvant (26). We have also identified anionic polysaccharide (A-PS) and A-PS-modified proteins in Pg OMVs as the immunodominant antigens. The results of ELISA coated with the whole cells of Pg showed the reactivity profile is very similar to that of Pgul in both mice (Fig. 1C and D; Fig. S2A and B) and beagles (Fig. 3 and Fig. 4A through D). The findings are clearly showing that Pg is antigenically similar to Pgul, and is also in line with a previous report, in which Pgul has virulence and immunologically similar to those of Pg (42). We assume that not only Pg but also Pgul utilize a type IX secretion system (T9SS) and decorate A-PS modified proteins, which are substrates of T9SS, on the outer membrane. Furthermore, the surface appearance of OMVs of three *Porphyromonas* species (Pg, Pgul, and Psal) was found to be rougher than those of other species (Td, Aa, EcN) using a high-resolution FE-SEM (Fig. S1). The increased roughness at the outermost surface is likely attributed to the presence of abundant cargo proteins of T9SS, which was in line with our previous findings in which the surface appearance of OMVs of a T9SS mutant was significantly smoother than those of the parental strain (26). In terms of Td OMV antigen, Veith et al. reported the serine protease known as dentilisin, proteins containing leucine-rich repeats, and several lipoproteins containing FGE-sulfatase domains were enriched in OMVs (43). Dentilisin is a well-characterized virulence factor of Td, which shows cytotoxic activity against human epithelial cells (44), adherence to fibrinogen (45), and degradation of cytokine (46). The major surface protein (Msp) of Td and the proteins comprising the PrtP lipoprotein protease complex are expressed *in vivo* and are immunogenic in humans (47). The identification of immunodominant antigens of Pg and Td in beagles is an important issue for future research.

Our previous findings in BALB/c mice suggest that IN vaccines against periodontal diseases are likely more suitable than injected vaccines that do not elicit mucosal immune responses (25), and the resultant salivary SIgA responses following IN immunization contribute to bacterial clearance in the oral cavity (24, 26). In the mouse model of the present study, a three-dose regimen by the homologous IN immunization (IN-IN-IN) with a series of trivalent OMV combinations strongly elicited SIgA responses in saliva, i.e., OMVs of Pg/Td/Psal, Pg/Td/Aa, and Pg/Td/EcNΔ*flhD* (Fig. 1C and D; Fig. S2). Since the Pg/Td/EcNΔ*flhD* OMVs induced the strongest SIgA responses in saliva of mice (Fig. 1C and D; Fig. S2), we chose and used the Pg/Td/EcNΔ*flhD* OMV vaccine for beagle experiments. However, the trivalent OMV intranasal administration to beagles did not induce SIgA responses well (Fig. 3). An attempt at subcutaneous booster at week 21 successfully enhanced not only serum IgM/G but also salivary SIgA production. The marked difference in immune responses between mouse and dog was probably due to the difference in the commensal microbiota between mouse and dog. It has been well-known that canines, unlike mouse, share common microorganisms on the mucosal surface with humans (20, 48). As we showed in Fig. 5E, periodontal pathobionts were frequently detected in beagles even at 18 weeks old (10 weeks after first immunization) by real-time PCR. We therefore hypothesize that immunological tolerance due to these bacteria as commensals in the oral cavity resulted in unresponsiveness to the IN immunization (Fig. 3). On the other hand, the SC booster strongly improved immune responses in both blood and various mucosal sites (Fig. 3 and 4). It may be true that the vaccination by the SC route activates the adaptive immune system network composed of antigen-presenting cells and memory T/B cells more directly than the vaccination via the IN route, so that the SC booster could evoke stronger activation of adaptive immune responses (Fig. 3 and 4). Taken together, the present study proposes the utility of the IN prime followed by the SC boost regimen as a rational option to elicit robust humoral immune responses in canines, and most probably in humans as well.

The primary objective of the current study was to induce a humoral immune response in young beagles following the OMV vaccine. Since the beagle study was initiated at 2 months old and terminated at 10 months old, it was too early to examine the vaccine efficacy against spontaneous development of periodontal disease in beagles. On the other hand, we also examined how the OMV vaccine affected oral microbiota. The salivary metagenome analysis showed that the trivalent Pg/Td/EcNΔ*flhD* OMV vaccine seemed to alter the composition of oral microbiota, while the numbers of two targets, Pg (or Pgul) and Td in vaccinated beagles were comparable to those in saline control beagles. One possibility for the negative result is that the vaccine schedule of the beagle experiment was not suitable to evaluate the vaccine effect. Given the high prevalence of Pg (or Pgul) and Td even in young beagles (Fig. 5E), the timing of immunization might be too late. In beagles aged 1 year or more than 3 years, the prevalence of clinical attachment loss greater than or equal to 1 mm was reported to be 20% or 84%, respectively (49). In addition, due to the intrinsic resistant property of periodontal biofilms against antibiotics and host immunity, a periodontal vaccine alone would not be effective in patients who have already had biofilms in the periodontal pockets. We therefore propose that the present vaccine schedule must be revised, e.g., immunization at a younger age or maternal vaccines to protect from the fetal stage. Alternatively, a combinational approach of vaccine and other periodontal treatment is presumably more effective than vaccination alone, e.g., professional mechanical tooth cleaning, antimicrobial therapy (prior to vaccination). On the other hand, another possibility for the negative results may be ineffectiveness of beagle antibodies induced by the OMV vaccine. In the functional analysis of beagle antibodies on the growth of Pg (Fig. 6), the serum samples of OMV-immunized beagles failed to inhibit the growth of Pg. On the other hand, serum samples of OMV-immunized beagles inhibited the growth of the gingipain-deficient Pg strain. The findings suggest that gingipains might inactivate the beagle’s immune system by degrading proteinaceous molecules such as protective antibodies and complements. Arastu-Kapur et al. have recently reported that oral administration of a gingipain inhibitor reduced the number of Pgul in the oral cavity of aged dogs (50). A therapeutic strategy targeting gingipains may be necessary along with the development of vaccines.

Periodontal diseases are the most prevalent oral diseases in humans and have serious health and economic burdens, greatly reducing quality of life (10). To overcome the problem, novel periodontal vaccines based on rational perspectives could reduce not only the prevalence of periodontal diseases but also the common risk factors with other non-communicable diseases, such as diabetes and cardiovascular diseases. In the present study, our trivalent OMV vaccine strategy by IN-prime/SC-boost regimen could elicit robust mucosal immune responses. However, the present beagle study has a limitation regarding the sample size, as well as the study period. Due to the relatively small sample size, it should be noted that there is a possible risk of over- or under-estimation of the association between intervention and outcome in this study. Therefore, the study should be expanded to larger veterinary clinical trials with longer follow-up, which would provide further evidence on the efficacy of the OMV vaccine. In the future, we are going to investigate whether the revised OMV-based vaccines can induce safe and effective immune responses in not only companion animals, but also primates, with a view to subsequent clinical trials with human subjects.

## SUPPLEMENTAL MATERIAL

Supplemental figures msphere.01033-24-s0001.pdf Figures S1 to S6.

Table S1 msphere.01033-24-s0002.xlsx Primers and fluorescent probes.

Table S2 msphere.01033-24-s0003.xlsx Identified OTUs increased in the groups of saline and OMVs.

ASM does not own the copyrights to Supplemental Material that may be linked to, or accessed through, an article. The authors have granted ASM a non-exclusive, world-wide license to publish the Supplemental Material files. Please contact the corresponding author directly for reuse.
