# Supplementary material for: Assessment of periodontitis vaccine using three different bacterial outer membrane vesicles in canine model
Source: mSphere. 2025 Mar 18;10(4):e01033-24. doi: 10.1128/msphere.01033-24 (PMC12039265; doi:10.1128/msphere.01033-24)
Supplement: Supplemental figures — Figures S1 to S6. [file msphere.01033-24-s0001.pdf]

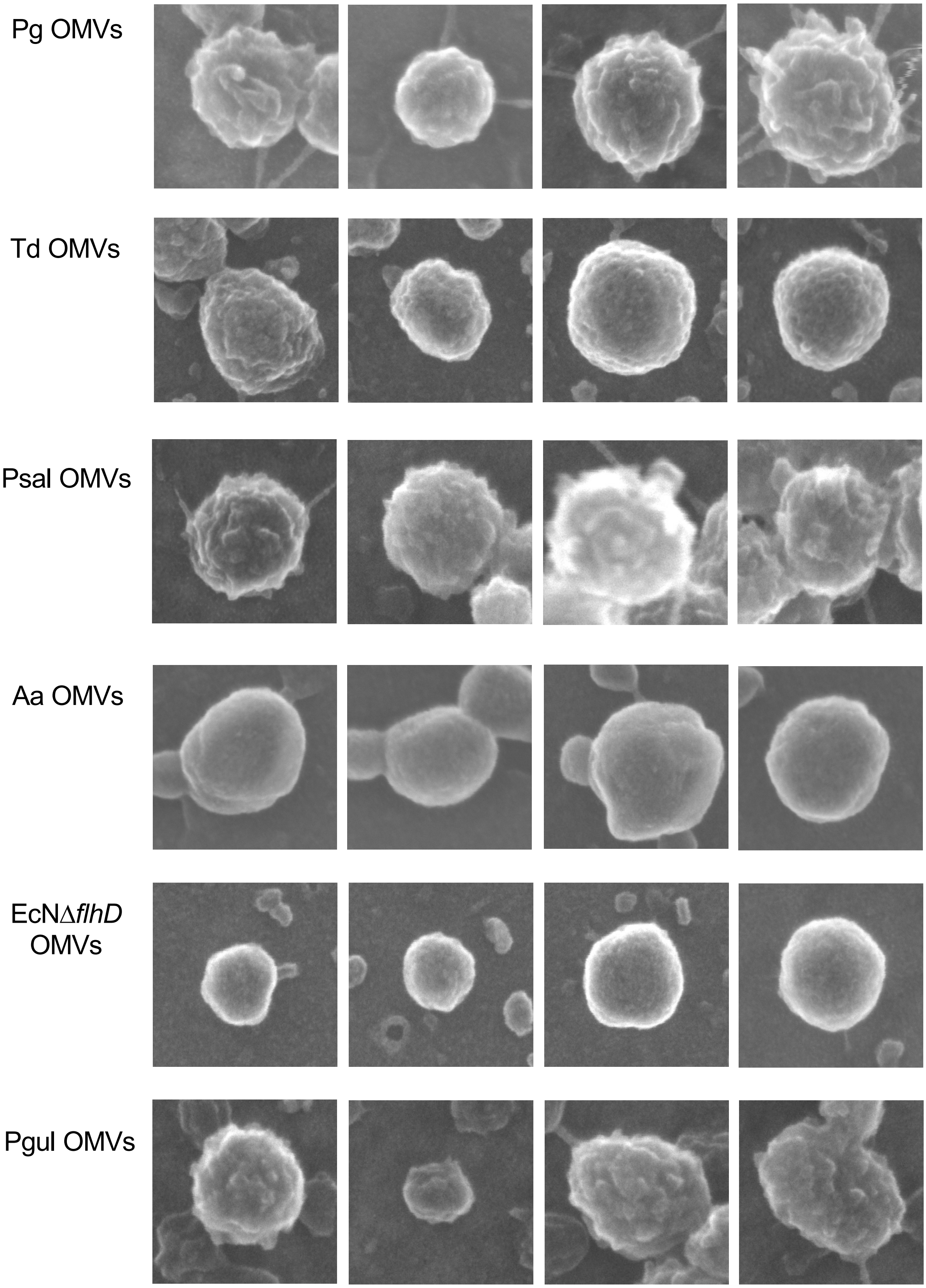


**Fig. S1**

**Fig. S1. Morphology of different OMVs**

The surface appearance of OMVs of six different species (Pg, Td, Psal, Aa, EcN, and Pgul) using FE-SEM. Shown are four randomly chosen OMVs. The area of each picture was 100 × 100 (x × y) $nm^2$.

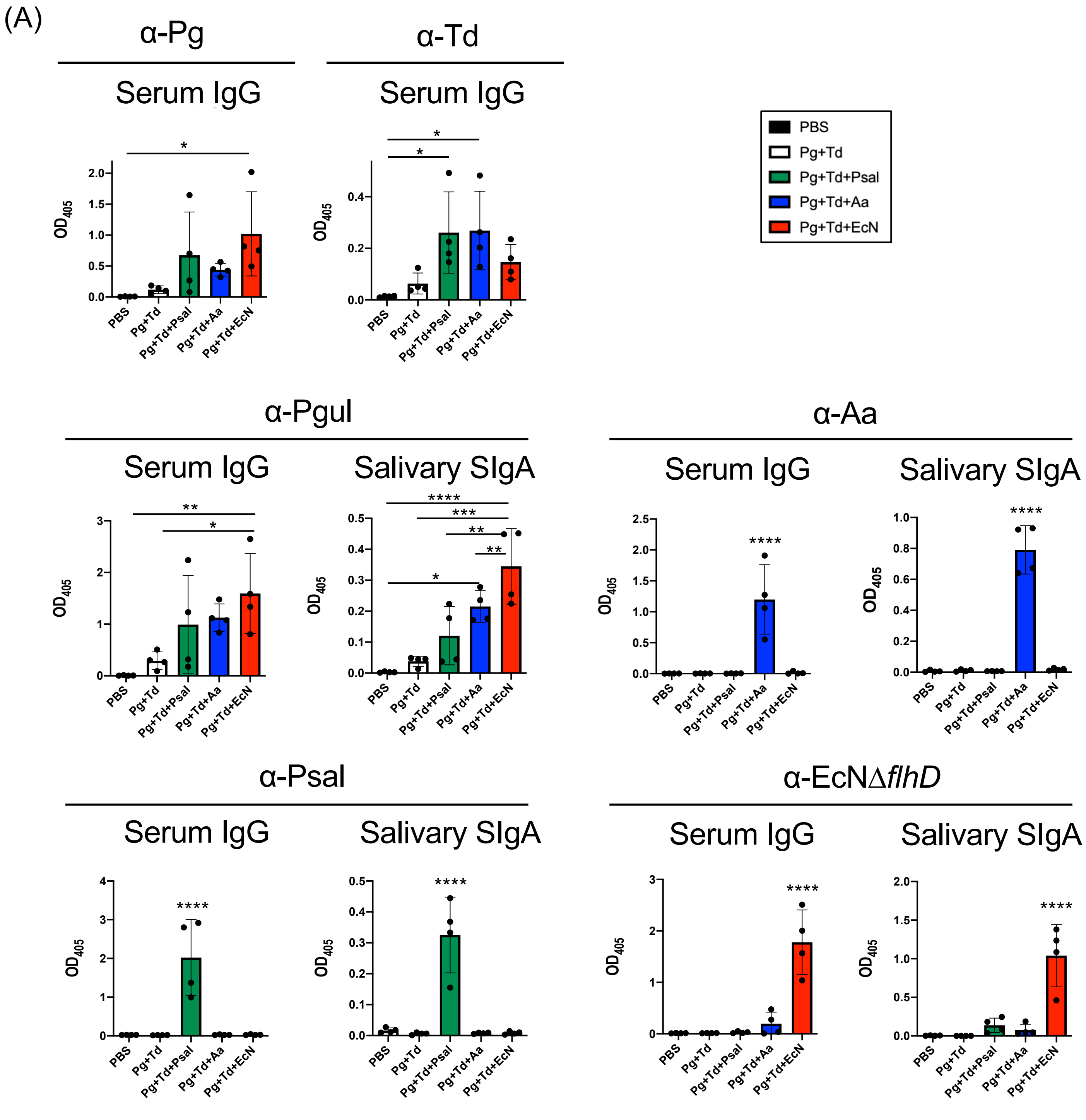
(A)
α-Pg
Serum IgG
α-Td
Serum IgG
PBS
Pg+Td
Pg+Td+Psal
Pg+Td+Aa
Pg+Td+EcN
α-PguI
Serum IgG
Salivary SIgA
α-Aa
Serum IgG
Salivary SIgA
α-Psal
Serum IgG
Salivary SIgA
α-EcNΔ*flhD*
Serum IgG
Salivary SIgA
$OD_{405}$

**Fig. S2**

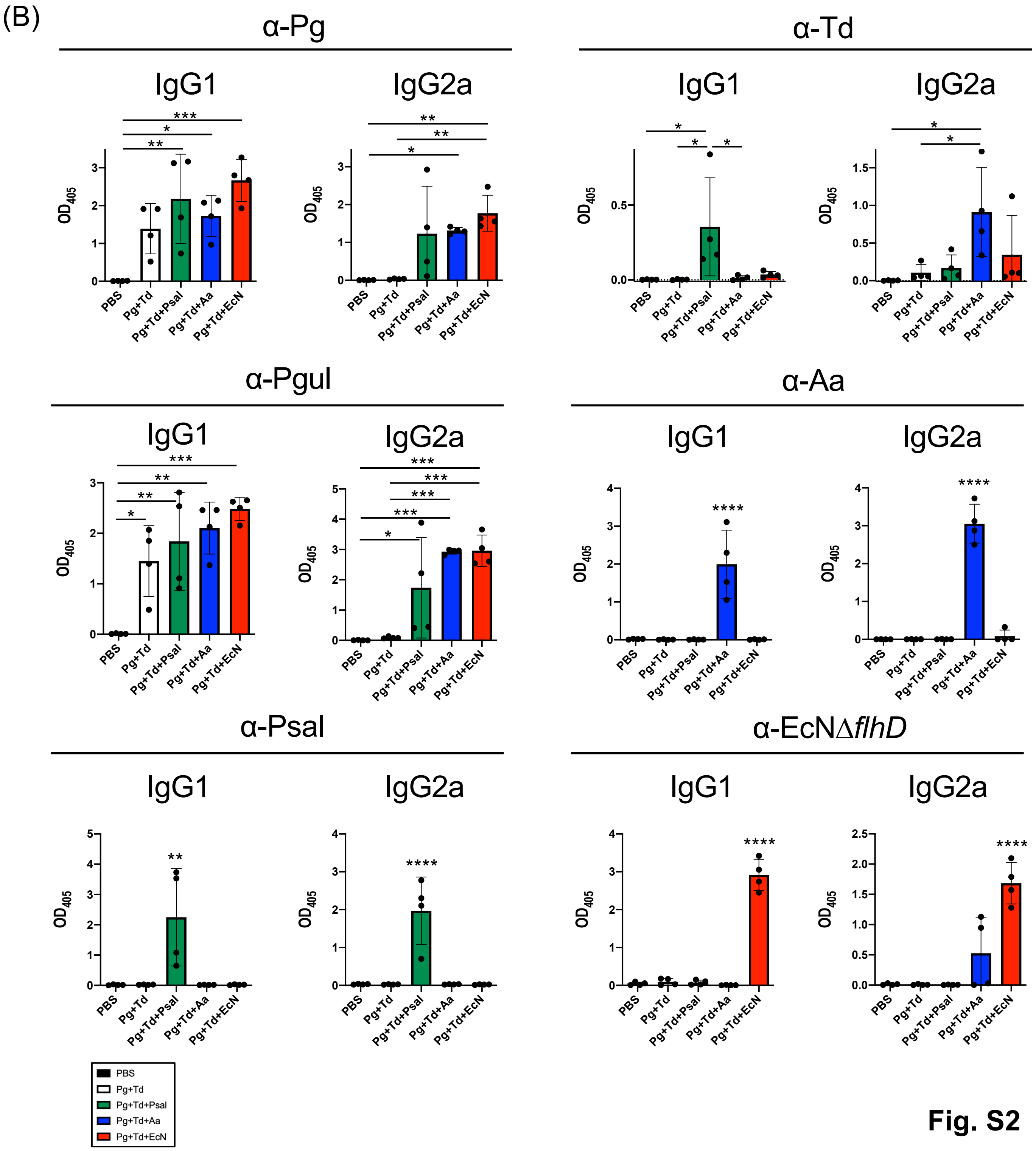


**Fig. S2**

**Fig. S2. Mucosal adjuvanticity of different bacterial species-derived OMVs in mouse model vaccinated with Pg OMVs and Td OMVs**

(A) Humoral immune responses (serum IgG and salivary SIgA) in mice after OMV vaccines. Serum and saliva samples were used at 1:1,000 and 1:100 dilutions, respectively, for whole-cell ELISA. The results of IgG ELISA are expressed as $OD_{405}$ values (mean ± SD) after a 15-min incubation (anti-Pg, Pgul, Aa, EcNΔ*flhD*) or a 120-min incubation (anti-Td, Psal) with AP substrate. The results of all IgA ELISA are expressed as $OD_{405}$ values (mean ± SD) after a 120-min incubation with AP substrate.

(B) Humoral immune responses (serum IgG1 and IgG2a) in mice after OMV vaccines. Both serum IgG1 (left panels) and IgG2a (right panels) were examined by whole-cell ELISA. Serum samples were used at 1:100 dilutions. The results are expressed as $OD_{405}$ values (mean ± SD) after a 30-min incubation (anti-Pgul), a 45-min incubation (anti-Pg, Aa), a 60-min incubation (anti-EcNΔ*flhD*), or a 90-min incubation (anti-Td, Psal) with AP substrate.

One-way ANOVA followed by Tukey's multiple comparison test was used for the statistical analysis. *$p < 0.05$. **$p < 0.01$. ***$p < 0.001$. ****$p < 0.0001$.

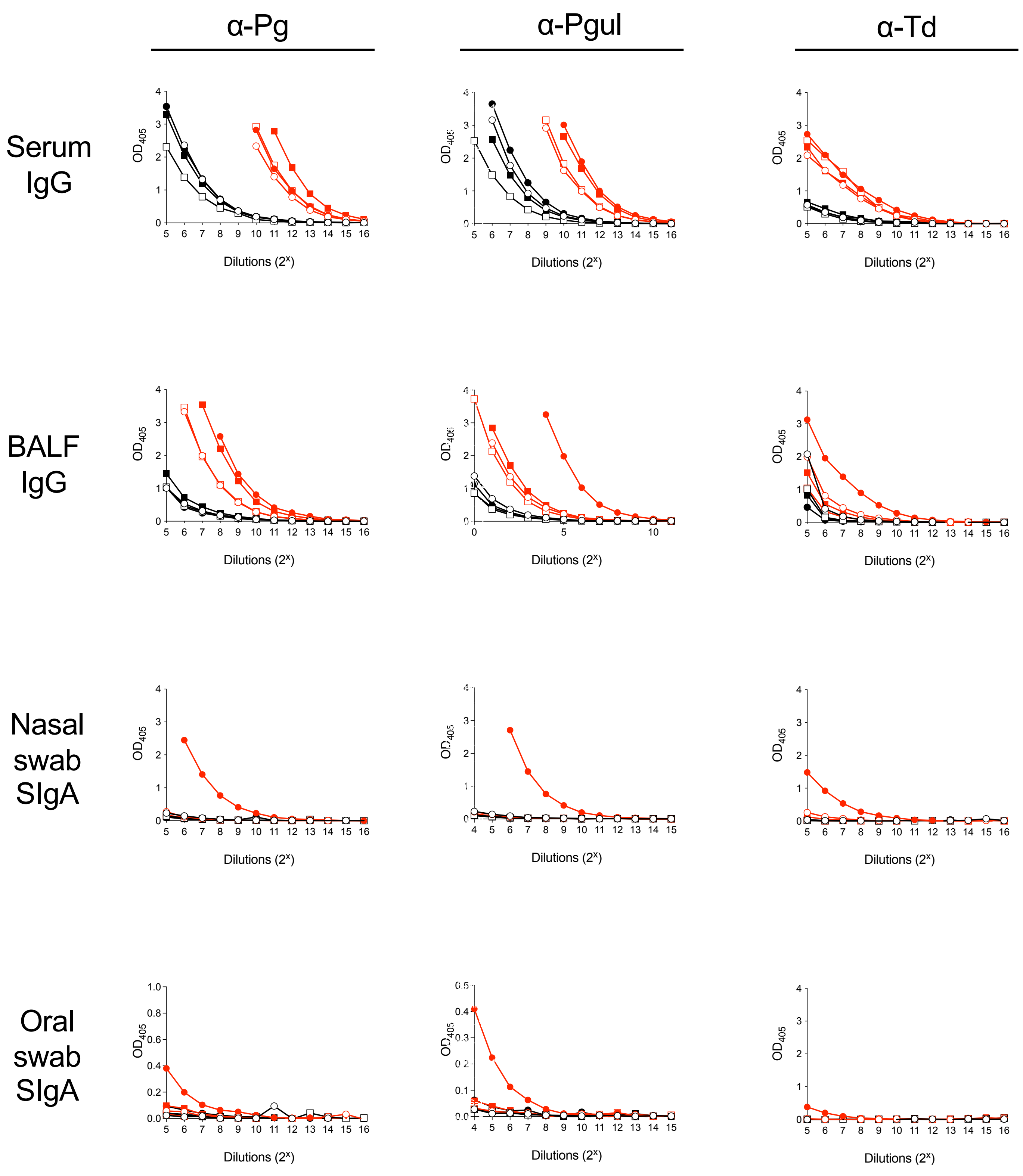


| Sex | Intervention |
|---|---|
| ○ Female | Saline-1 |
| ● Female | Saline-2 |
| □ Male | Saline-3 |
| ■ Male | Saline-4 |
| ○ Female | OMVs-1 |
| ● Female | OMVs-2 |
| □ Male | OMVs-3 |
| ■ Male | OMVs-4 |

**Fig. S3. Pg, Pgul, and Td-specific antibody responses in beagles after euthanization**

Pg-, Pgul-, and Td-specific antibody responses (serum IgG, BALF IgG, nasal swab IgA, and oral swab IgA) were examined by ELISA coated with whole cells of the respective bacteria. Shown are the dose-response curves obtained by 2-fold serially diluted samples in whole-cell ELISA. X-axes showed the sample dilutions ($log_2$). Y-axes showed $OD_{405}$ values after a120-min incubation with AP substrate.

**Fig. S3**

# (A) Components increased in saline group

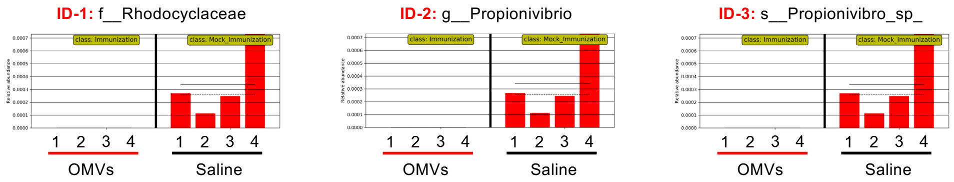


# (B) Components increased in OMVs group

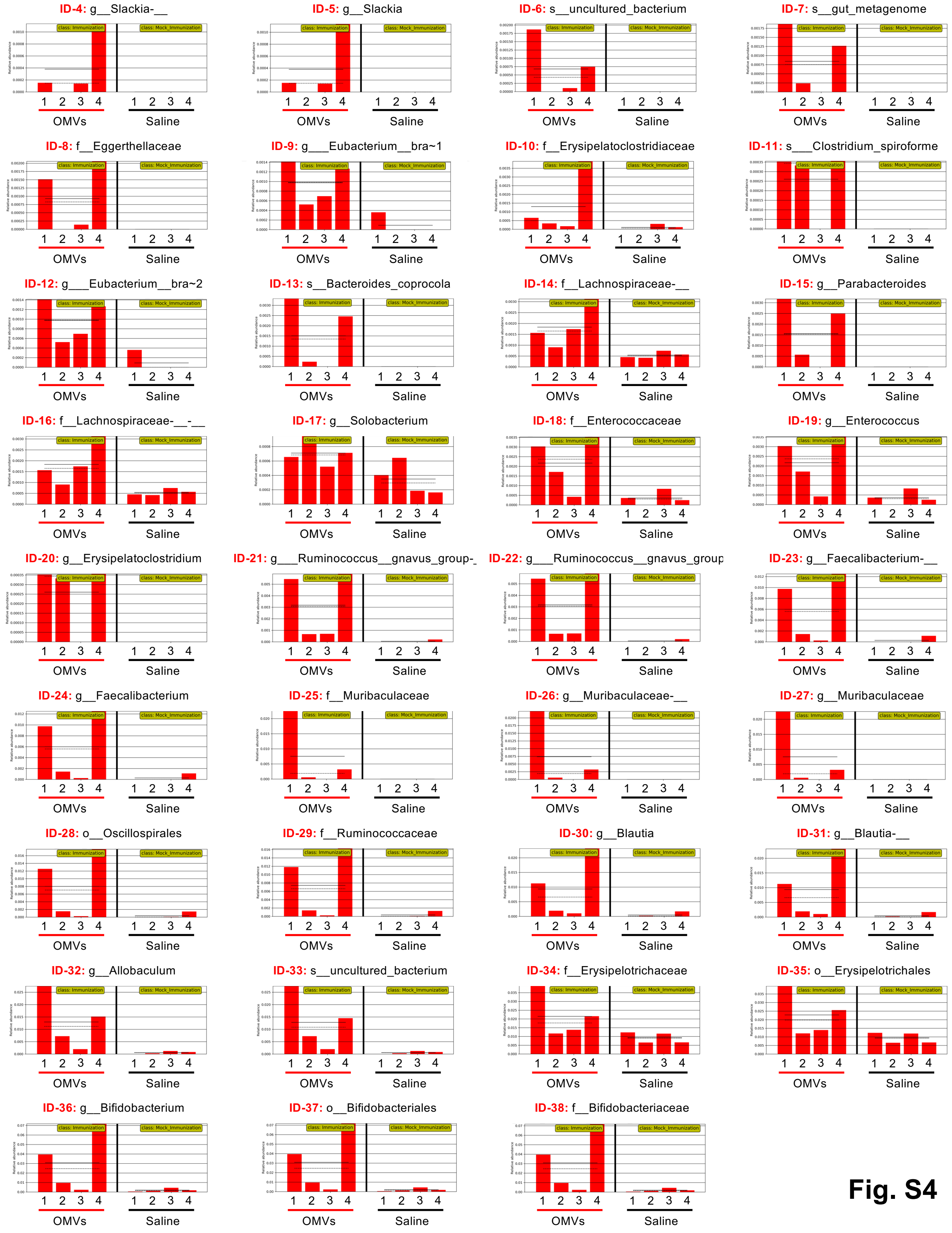


**Fig. S4**

**Fig. S4. Increased genus and higher levels in saliva of mock- and OMV-immunized groups.**

Statistically significant difference in abundance of some genus and the higher levels between OMVs and saline groups at week 32 was detected by comparative analyses of the taxonomic composition of the salivary microbiome. (A) Three components increased in saline group than OMVs group ($p < 0.05$, in all panels). (B) Thirty-five components increased in OMVs group than saline group ($p < 0.05$, in all panels). ANOVA followed by the Dunnett's multiple comparison test was used for the statistical analysis. OTUs are indicated on the top of each figure. See also Fig. 5G and Table S2.

Feces (OMVs vs Saline)

Components increased in saline group

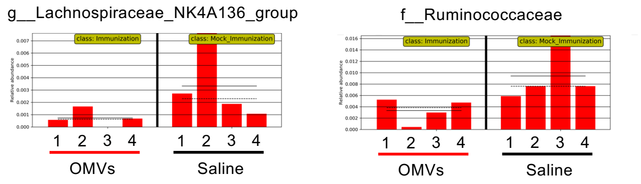


**Fig. S5**

**Fig. S5. Increased genus and higher levels in feces of mock- and OMV-immunized groups.**
Statistically significant difference in abundance of some genus and the higher levels between OMVs and saline groups at week 32 was detected by comparative analyses of the taxonomic composition of the feces microbiome. Only two components increased in saline group than OMVs group ($p < 0.05$, in all panels). No components increased in OMVs group than saline group. One-way ANOVA followed by the Dunnett’s multiple comparison test was used for the statistical analysis.

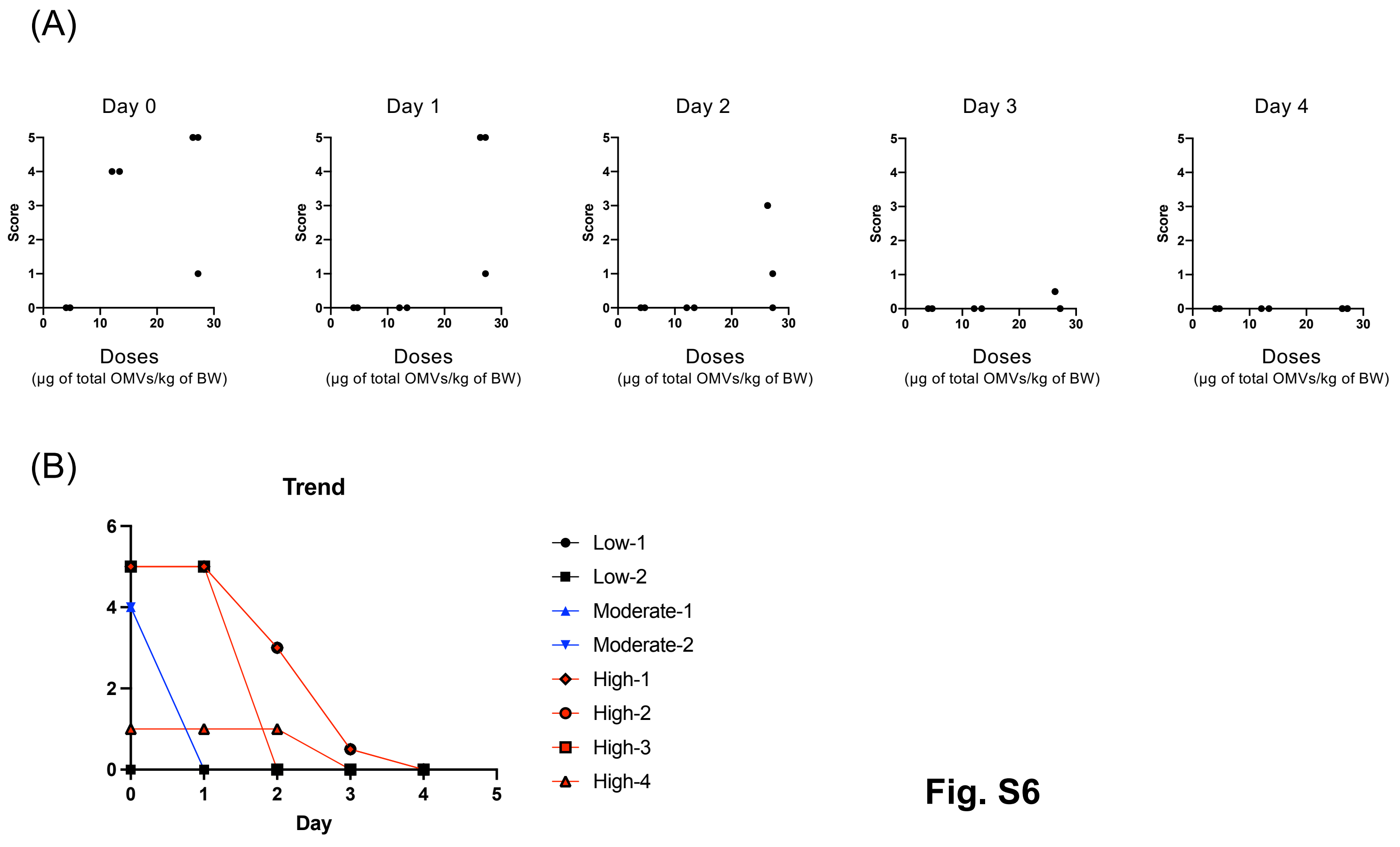


**Fig. S6**

**Fig. S6. Safety evaluation by adverse reaction scores in beagles**
The results of the evaluation of five parameters shown in Table 1 for general conditions were graphed. Y-axes show the scores of general conditions. X-axes of (A) show the vaccine doses (µg of total OMVs/kg of body weight) from day 0 to day 4. A time-course diagram for each subject in (A) is shown in (B).
